# Cognitive Deficits Are Attenuated in Neuroglobin Overexpressing Mice Exposed to a Model of Obstructive Sleep Apnea

**DOI:** 10.3389/fneur.2018.00426

**Published:** 2018-06-05

**Authors:** Deepti Nair, Vijay Ramesh, David Gozal

**Affiliations:** ^1^Section of Sleep Medicine, Biological Sciences Division, Department of Pediatrics, Pritzker School of Medicine, The University of Chicago, Chicago, IL, United States; ^2^Atlantic Health System, Morristown, NJ, United States; ^3^Biomedical Research Institute of New Jersey, Cedar Knolls, NJ, United States

**Keywords:** intermittent hypoxia, neuroglobin, sleep apnea, oxidative stress, cognitive impairment, neuroprotective

## Abstract

**Background:** Obstructive sleep apnea (OSA) is a highly prevalent disease manifesting as intermittent hypoxia during sleep (IH) and is increasingly recognized as being independently associated with neurobehavioral deficits. These deficits may be due to increased apoptosis in the hippocampus and cerebral cortex, as well as increased oxidative stress and inflammation. It has been reported that neuroglobin (Ngb) is upregulated in response to hypoxia-ischemia insults and exhibits a protective role in ischemia-reperfusion brain injury. We hypothesized that transgenic overexpression of Ngb would attenuate spatial learning deficits in a murine model of OSA.

**Methods:**Wild-type mice and Ngb overexpressing male mice (Ngb-TG) were randomly assigned to either IH or room air (RA) exposures. The effects of IH during the light period on performance in a water maze spatial task were assessed, as well as anxiety and depressive-like behaviors using elevated plus maze (EPM) and forced swim tests. Cortical tissues from all the mice were extracted for biochemical studies for lipid peroxidation.

**Results:**Ngb TG mice exhibited increased Ngb immunoreactivity in brain tissues and IH did not elicit significant changes in Ngb expression in either Ngb-TG mice or WT mice. On a standard place training task in the water maze, Ngb-TG mice displayed preserved spatial learning, and were protected from the reduced spatial learning performances observed in WT mice exposed to IH. Furthermore, anxiety and depression levels were enhanced in WT mice exposed to IH as compared to RA controls, while alterations emerged in Ngb-TG mice exposed to IH. Furthermore, WT mice, but not Ngb-TG mice had significantly elevated levels of malondialdehyde in cortical lysates following IH exposures.

**Conclusions:**In a murine model of OSA, oxidative stress responses and neurocognitive and behavioral impairments induced by IH during sleep are attenuated by the neuroprotective effects of Ngb.

## Introduction

A substantial body of evidence indicates that sleep disordered breathing (SDB) is a substantial and prevalent health problem in both the adult and pediatric populations. Indeed, a conservative estimate would suggest that around 5% of children and up to 15–35% of adults in the general population suffer from the most common and serious form of SDB, namely obstructive sleep apnea (OSA). OSA is characterized by recurrent episodes of upper airway obstruction or partial obstruction during sleep that result in intermittent hypoxia (IH) and hypercapnia, sleep disruption, and increased intrathoracic pressure swings due to respiratory effort aimed at opening the collapsed airway. OSA has now been recognized to increase the risk of gray matter losses and accompanying neuropsychological impairments in humans. These impairments display heterotopic distribution, with frontal cortex and hippocampal neurons being particularly sensitive to IH, both *in vivo* and *in vitro* (1–3). Further, rodent models have conclusively supported the hypothesis that chronic exposures to IH during sleep result in significant spatial learning deficits, as well as with increased risk of apoptotic processes in neurons within susceptible brain regions such as the hippocampus and cerebral cortex, and that such deficits involve oxidative stress (1, 4, 5).

Neuroglobin (Ngb) is a hypoxia inducible factor 1α (HIF 1α) regulated globin, which was first identified as a protein that is mainly expressed under hypoxic or ischemic conditions in regions corresponding to both the central and peripheral nervous systems (6–10). Additional studies have confirmed that Ngb plays a neuroprotective role both *in vitro* (6) and *in vivo* (9, 11, 12), most likely via a ROS scavenging function (13). Ngb overexpressing mice generated in our laboratory have clearly shown that the expression patterns of Ngb include neuronal populations within the hippocampal formation, and that Ngb constitutive overexpression in the transgenic mice does not alter the endogenous antioxidant system. Studies conducted with the Ngb overexpressing mice on a mouse model of ischemia-reperfusion injury markedly reduced both the production of ROS and reactive nitrogen species (RNS), as well as lipid peroxidation in the CA1 region, all of which resulted in reduced CA1 neuronal injury (9). Furthermore, Ngb also scavenges toxic reactive species, such as nitric oxide, peroxynitrites and hydrogen peroxide (14). In mice, CNS administration of an Ngb antisense oligodeoxynucleotide augmented ischemic infarct size, and worsened neurological functional outcomes after focal ischemia, while adeno-associated virus-mediated Ngb over-expression ameliorated the extent of brain injury (6). A transgenic mouse model that constitutively over-expresses Ngb yielded similar findings after brain ischemia, with the cerebral infarct volume being reduced by ~30% (15). Ngb overexpression also attenuated oxidative stress markers in a mouse MCAO model (16), thereby pointing to cellular defense mechanisms being recruited against oxidative stress during hypoxia/ischemia insults. However, the neuroprotective role of Ngb in the context of the IH patterning that characterizes OSA remain largely undefined. Here, we hypothesized that Ngb would afford a protective role against IH-induced cognitive deficits in a murine model of OSA. To this effect, we examined whether transgenic neuroglobin overexpressing (Ngb-TG) mice displayed improved cognitive function in a spatial task and reduced anxiety in the elevated plus maze (EPM) following prolonged exposures to IH during the rest period, since anxiety, depression and memory deficits are readily apparent in a significant proportion of patients with sleep apnea (17–19).

## Materials and methods

### Animals

A complementary DNA (cDNA) encoded with human wildtype Ngb was synthesized using a modification of recursive PCR strategy. Neuroglobin cDNA was then sub-cloned into an expression vector pcDNA3.1 (pcDNA3.1-Ngb). A BamH1- Xho1 fragment from the pcDNA3.1-Ngb was further sub-cloned into a pUB6 plasmid with the human ubiquitin C promoter to achieve ubiquitous expression of the transgene. The Ngb transgenic mice were then generated to incorporate and overexpress the human wild-type Ngb at the transgenic core facility at the University of Louisville (9). The first generation of Ngb overexpressing transgenic mice was then backcrossed with the C57/B6 strain for at least 10 generations and all the mice were genotyped to confirm continued expression of the transgene. Neuroglobin mRNA and protein expression in the brain were assessed at the age of 2 months, i.e., the age at which the mice were used for the study, and showed increased expression as previously reported (9). Furthermore, we have previously shown that sustained hypoxia, but not IH, elicit increased expression of Ngb (7). Male C57BL/6J (WT) mice were purchased from Jackson Laboratories (Bar Harbor, Maine) at 5 weeks of age, and housed at the animal facility at the University of Chicago until they attained 8 weeks of age at which time they were randomly paired to serve as controls for the Ngb transgenic mice. Animals were housed in a 12-h light/dark cycle-controlled room (lights on at 7:00 a.m. till 7:00 p.m.) with ambient temperature being kept at 26 ± 1°C. Male mice were placed in groups of four mice in standard clear mouse polycarbonate cages and were allowed unrestricted access to standard chow food and water. All behavioral experiments were conducted during the light period (between 9:00 a.m. and 12:30 p.m.). Mice were randomly allocated to either IH or room air (RA) exposures. The experimental protocols were approved by the Institutional Animal Use and Care Committee at the University of Chicago (protocol # 72043) and are in close agreement with the National Institutes of Health *Guide in the Care and Use of laboratory animals*. All efforts were made to minimize animal suffering and to reduce the number of animals used.

### Intermittent hypoxia exposures

**Figure 1:**
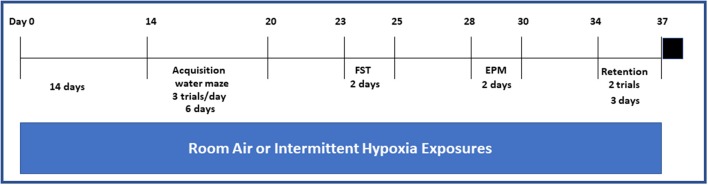
Schematic diagram on the sequence of exposures to either IH or RA and behavioral experiments and in both wild type and Ngb TG mice. Black square on the far end right of the figure indicates euthanasia. Please note that exposures to either IH or RA were continued throughout the duration of the protocol.

Animals were exposed to the desired environmental profile in commercially available Plexiglas chambers (30 × 20 × 20 in; Oxycycler model A44XO, BioSpherix, Redfield, NY) operated under a 12-h light-dark cycle (7:00 a.m. to 7:00 p.m.) (Figure 1) where the ambient temperature was kept at 26°C for 14 days prior to the actual behavioral testing. The intermittent hypoxia exposure protocols were implemented as described previously (5, 20–24). The oxygen concentration was continuously measured by an O_2_ sensor analyzer linked online to a servo-controlled system and oxygen and nitrogen gas flow was changed by a computerized system controlling the gas outlets, such as to generate either a cyclical pattern of 5.7 and 21% oxygen every 90 s (IH) during the sleep cycle or 21% oxygen throughout (RA). The nadir O_2_ concentration in the chamber was 5.7%, with a mean concentration over the cycle of 10.2%. For the remaining 12 h, oxygen concentration was kept at 21%. The cumulative arterial blood gases at various time points during the alternating 10% O_2_-room air 90 sec cycle confirmed the anticipated alternation of moderate hypoxemia with normoxemia, such that calculated oxyhemoglobin saturations ranged from 62 to 70%, and were clearly within the range of those recorded in moderately severe OSA patients.

### Western blotting

Brain tissues were homogenized by standard procedures. Homogenate proteins (50 mg) were heated for 10 min at 90°C, loaded onto 18% PAGE gels, then transferred electrophoretically onto nitrocellulose membranes. Membranes were incubated overnight at 4°C with the primary antibody (anti-Ngb, diluted 1:1,000, Biovendor), as previously described (9). Neuroglobin protein bands were detected with secondary antibodies and visualized by chemiluminescence, followed by stripping and re-blotting with β-actin antibody (Sigma, St Louis, MO, USA). After densitometric measurements were performed (Molecular Dynamics, Sunnyvale, CA, USA), Ngb blots were then normalized to β-actin. Data were expressed as fold increase of corresponding wild type controls.

## Behavioral studies

### Spatial learning and memory

Morris water maze spatial learning task and testing routines were conducted using a white circular pool with water being maintained at a temperature of 21°C. A Plexiglas escape platform was positioned 1 cm below the water surface which was opacified with white tempura paint. Fixed extra-maze cues surrounded the maze in surrounding positioned curtains and were readily visible to the mice while located in the pool. Maze performance was recorded by a video camera placed immediately above the pool, and interfaced with a video tracking system (HVS Imaging, Hampton UK) (5, 21, 25). A standard place-training reference memory task was conducted on mice in the water maze system following their exposure to 14 days of either IH or RA. Exposures were continued throughout the duration of the spatial maze training and testing. During the day before the beginning of place learning, mice were brought and habituated to the water maze during a free swim exposure aimed at reducing the potential stress associated with such experience. Place learning performance was then evaluated over six consecutive days using a regularly spaced training regimen that has been demonstrated to induce optimal learning in mice (26). Each place-training session consisted of three similar trials separated by a 10-min inter-trial interval. On any particular daily session, each animal was placed into the pool from one of four random start points based on standard coordinates. Mice were then allowed to swim in the pool for up to 90 s and to escape to the submerged platform, at which time mice were allowed to remain for 15 s on the platform. The platform was then removed 24 h after the final training session to appraise measures of spatial bias (probe trial) (25). To assess the performance in the water maze, mean escape latencies and swim distance were analyzed, quantified and computed, both automatically by the software as well as manually by an independent observer (VR) who was blinded to the experimental conditions and animal strain.

#### Reference memory

Retention spatial bias tests were carried out 14 days after acquisition of the spatial task (Figure 1). In the retention evaluation sessions, performance in a single session (two trials) was assessed, and the mean average performance of the two trials was calculated. Mice continued their exposures to the corresponding IH or RA exposures during the interval between task acquisition and spatial bias retention testing.

### Elevated plus maze (EPM)

The EPM was used to assess anxiety behaviors. The apparatus consists in an elevated cross formed by two open arms) and two closed arms made of transparent Plexiglas radiating from a central platform to form a plus-sign. The device was situated 51 cm above the floor. A 60-w light was placed above the apparatus and the test was videotaped by a camera overhead. Behaviors during each test were recorded by the video camera that was positioned above the maze and the number of entries into open and closed arms and the time spent on each arm were registered (Noldus Ethovision, Leesburg, VA) (25). The open arms are considered by mice as a threatening area. Animals were placed into the central area facing one open arm and allowed to explore the maze for 5 min. The following parameters were scored: (a) Percent time spent in open and closed arms; (b) number of entries to closed arms; (c) Time spent in the center. An arm entry was defined as the entry of all four feet into either one of the closed arm. The percentage of time spent in the open arm is commonly used as a measure of anxiety, while the time spent on the center platform of the maze and the closed arm entries are deemed to reflect anxiety-like behaviors in mice. Of note, the maze was cleaned with 30% ethanol between trials to remove any antecedent odor cues.

### Forced swimming test (FST)

Mice were individually placed and forced to swim in an open cylindrical container (diameter 14 cm, height 20 cm), with a depth of 15 cm of water at 25 ± 1°C. The immobility time, defined as the absence of escape-oriented behaviors, was scored for a total period of 6 min, as previously described (25). Each mouse was deemed as being immobile when it ceased struggling or swimming, and instead remained floating motionless in the water, making only those movements necessary to keep its head above water. The average percentage immobility was recorded and computed by a blinded experimenter.

### Biochemical studies

Upon completion of all the aforementioned behavioral experiments (5), the mice were returned back to their respective conditions IH or RA until they were sacrificed at 7:00 p.m. by cervical dislocation and the brains were immediately dissected under dry-ice and frontal cortical tissues were extracted. The tissues were flash frozen in liquid nitrogen and stored in −80°C until assayed.

### Lipid peroxidation assay

MDA, which is an index measure of lipid peroxidation was assayed in frontal brain cortex samples using a commercially available assay kit (Bioxytech MDA-586; OxisResearch, Portland OR) according to the manufacturer's instructions. Cortical tissues were homogenized in 20 mM phosphate buffer (pH 7.4) containing 0.5 mM butylated hydroxytoluene to prevent sample oxidation. Lysates were then centrifuged at 1,000 *g* for 10 min, and 200 μL aliquots of the supernatants were used for the assay. A standard curve was used to determine the absolute concentration. Values were standardized to micrograms of protein for each of the individual samples.

### Statistical analysis

Both untransformed and normalized data were analyzed using the GraphPad Prism 5.01 (GraphPad software Inc., San Diego, CA,USA). Mean escape latencies and pathlengths were analyzed by analysis of variance repeated-measures and used to measure spatial acquisition and retention performances in the water maze. Tukey's *post-hoc* tests were used as appropriate. Similar statistical approaches were used to compare probe trials, reference memory tests, as well as EPM and FST results. Comparisons between the WT and Ngb transgenic overexpressing groups in room air and intermittent hypoxia conditions were computed by using analysis of variance procedures, followed by one-way multiple comparison *post-hoc* tests. Statistical significance was considered at *P* ≤ 0.05.

## Results

### Neuroglobin overexpression attenuates intermittent hypoxia-induced cognitive deficits in the morris water maze

**Figure 2:**
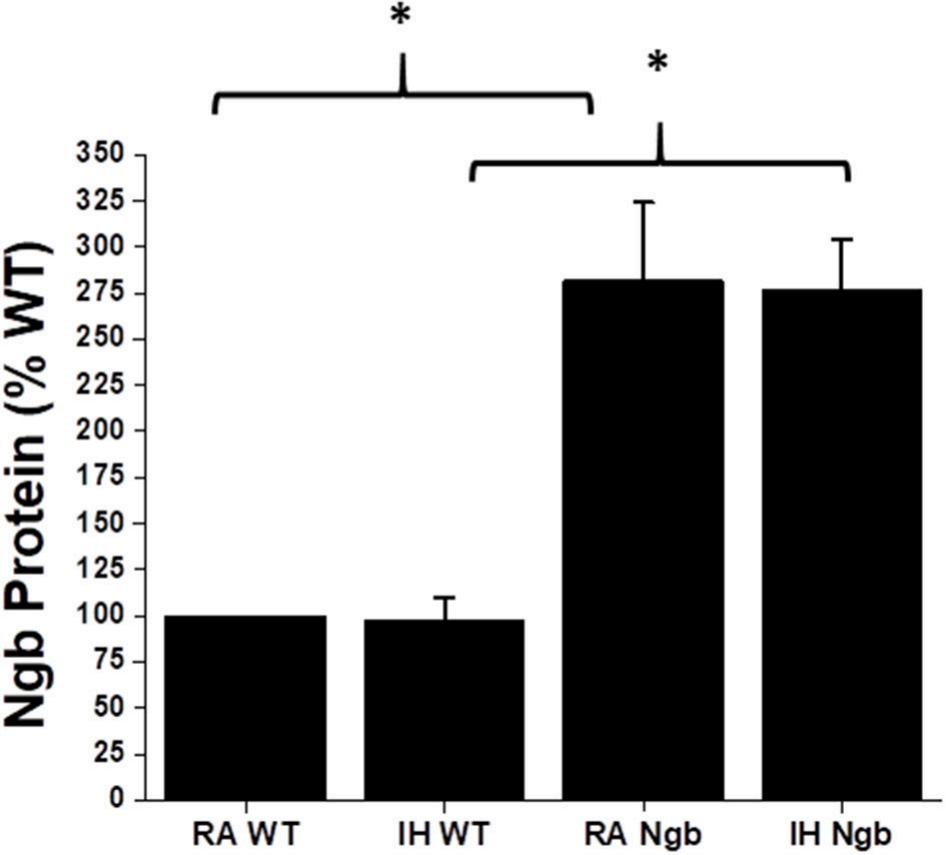
Ngb protein expression in brain tissues in wild type (WT) and Ngb TG mice exposed to room air or IH. Although Ngb TG mice exhibit significantly increased expression of Ngb protein, IH did not induce significant changes in Ngb expression in either WT or Ngb TG mice. (^*^p-value < 0.01; *n* = 6/group).

As previously reported (7), IH did not result in any significant changes in Ngb expression in WT mice (Figure 2). Similarly, although Ngb transgenic mice had significantly elevated expression of this protein in the brain, IH exposures did not induce measurable changes in Ngb expression (Figure 2).

**Figure 3:**
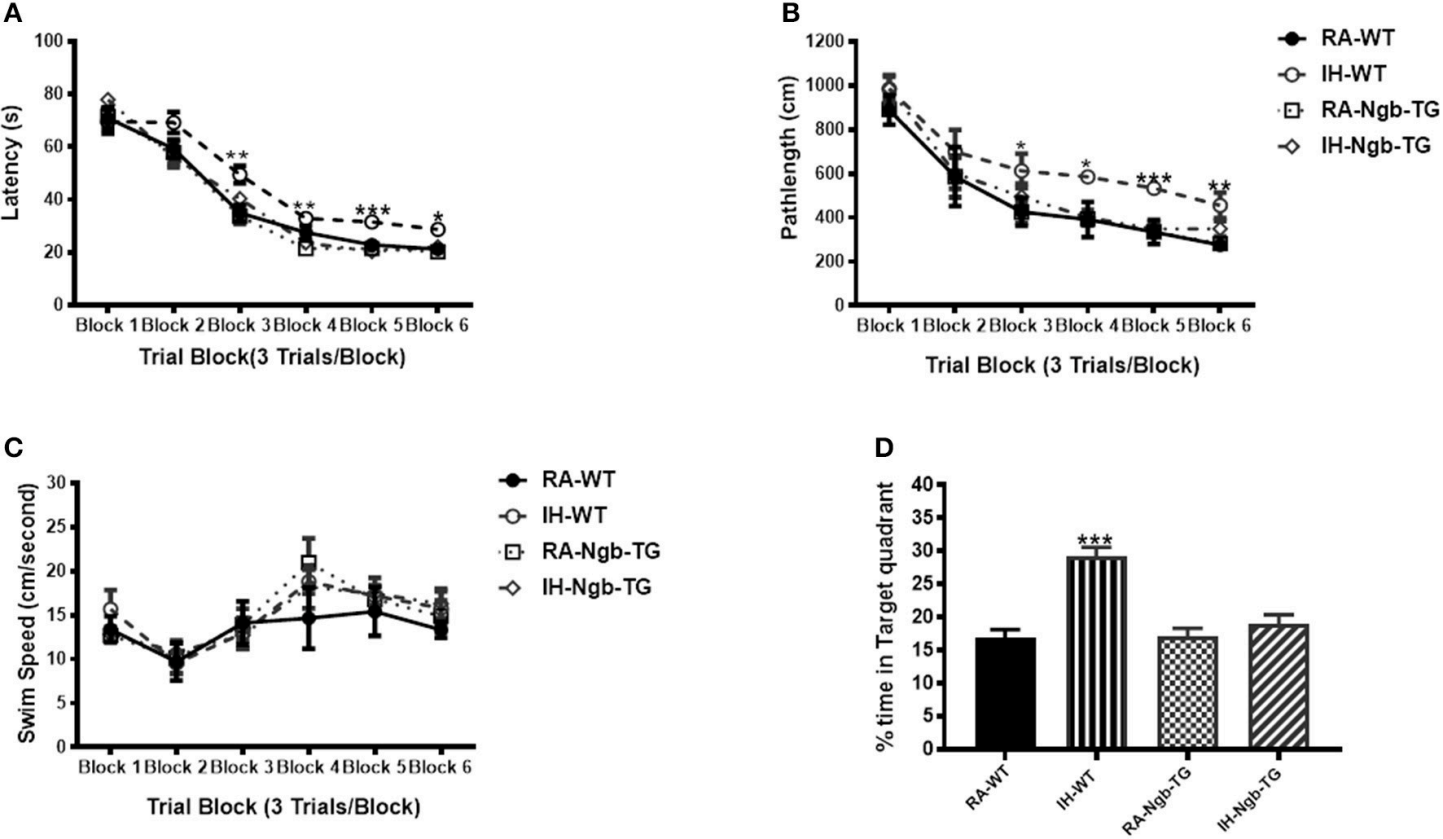
Ngb-TG mice exposed to IH do not exhibit deficits in spatial task acquisition and retention in the Morris water maze. **(A,B)** Mean latencies (s) and pathlength (cm) to locate the submerged platform during spatial task training in WT and Ngb-TG exposed to either intermittent hypoxia (IH) or to room air (RA) (*n* = 14 per group). **(C)** Swim speed **(D)** Mean percentage time in the target quadrant during probe trial after completion of water maze testing in either WT and Ngb-TG exposed to IH or maintained in RA. (*n* = 14/experimental group; ^*^P < 0.05, ^**^P < 0.001, ^***^P < 0.0001 vs. RA-WT).

**Figure 4:**
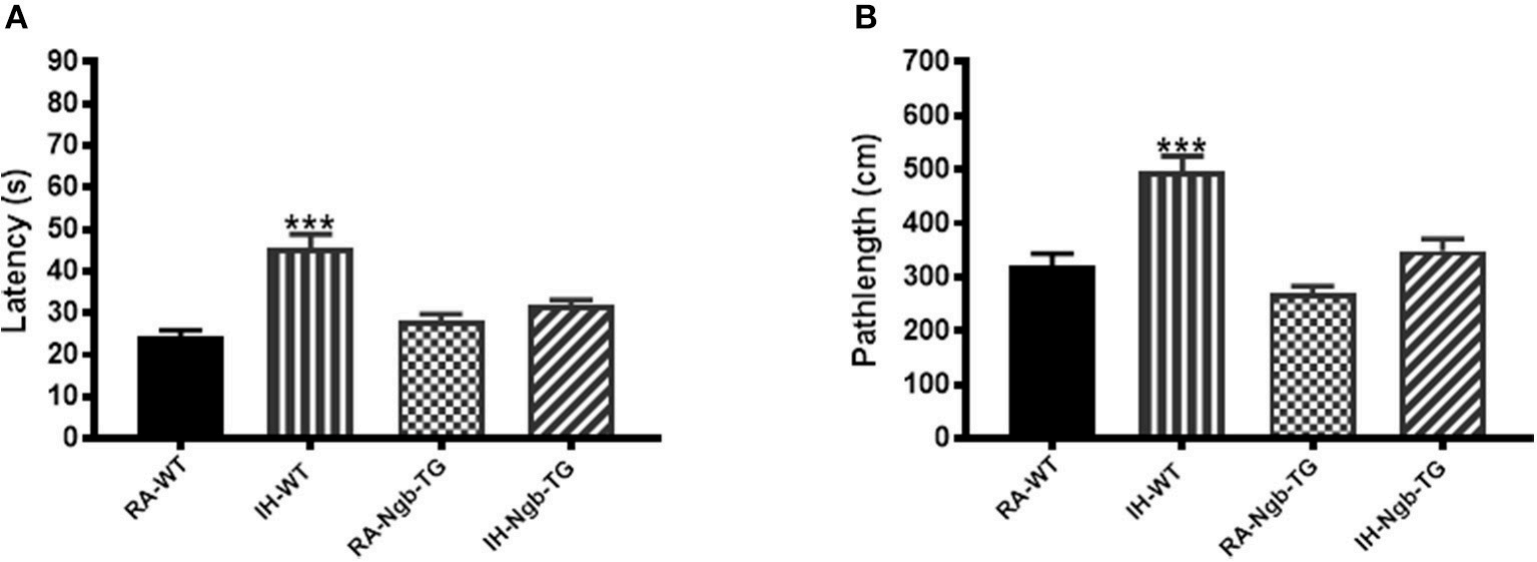
Ngb-TG mice exposed to IH do not exhibit deficits in spatial task retention in the Morris water maze. **(A)** Mean latencies (s) and **(B)** pathlength (cm) to locate the submerged platform location during retention trials in WT and Ngb-TG either exposed to intermittent hypoxia (IH) or maintained in room air (RA) in the Morris water maze. (*n* = 14/experimental group; ^***^P < 0.0001 vs. RA-WT).

To evaluate the effect of Ngb overexpression on cognitive function deficits induced by IH during the rest period, a standard Morris Water maze protocol was used. In the acquisition trials, WT mice exposed to RA (RA-WT) progressively and incrementally learned the location of the hidden platform, which was apparent by increasingly shorter latencies and shorter pathlengths during the test period (Figures 3A,B). However, exposures of WT mice to IH (IH-WT) for 14 days led to longer latencies and pathlengths to acquire the location of the hidden platform when compared to RA-WT, RA-Ngb-TG and IH-Ngb-TG animals (*n* = 14 per experimental condition; Figures 3A,B). In this behavioral procedure, the animal must acquire the location of a hidden platform using the distal surrounding cues, even as the starting position is randomly changed at each trial. Both latency and pathlength analysis were calculated for each block, the latter consisting of an average of 3 trials. Latency analysis of the blocks revealed significant changes during blocks 2 (*F* = 3.585; *p* < 0.0221), 3 (*F* = 6.406; *p* < 0.0012), 4 (*F* = 5.266; *p* < 0.0038), 5 (*F* = 10.28; *p* < 0.0001), and 6 (*F* = 5.337; *p* < 0.0035). There were no significant differences in Block 1. Pathlengths analysis for each the blocks revealed significant differences in pathlengths during blocks 3 (*F* = 4.975; *p* < 0.0051), 4 (*F* = 5.023; *p* < 0.0049), 5 (*F* = 8.822; *p* < 0.0001), and 6 (*F* = 5.143; *p* < 0.043), with no significant differences in blocks 1 and 2. There were no significant differences in swim speed across all experimental groups, indicating the absence of any detectable motor deficits (Figure 3C). In the probe-trial test, one-way ANOVA revealed a significant effect of treatment (IH vs. RA: *F* = 19.96; *p* < 0.0001). The magnitude of impairments was significantly greater in IH-WT mice (Figure 3D). In the reference memory tests, IH-WT mice exhibited significant deficits in spatial retention in both latency (*F* = 20.69; *p* < 0.0001) and pathlength (*F* = 20.31; *p* < 0.0001). However, the IH-Ngb-TG mice performed similarly to corresponding controls, i.e., RA-Ngb-TG (Figures 4A,B).

### Elevated plus maze

**Figure 5:**
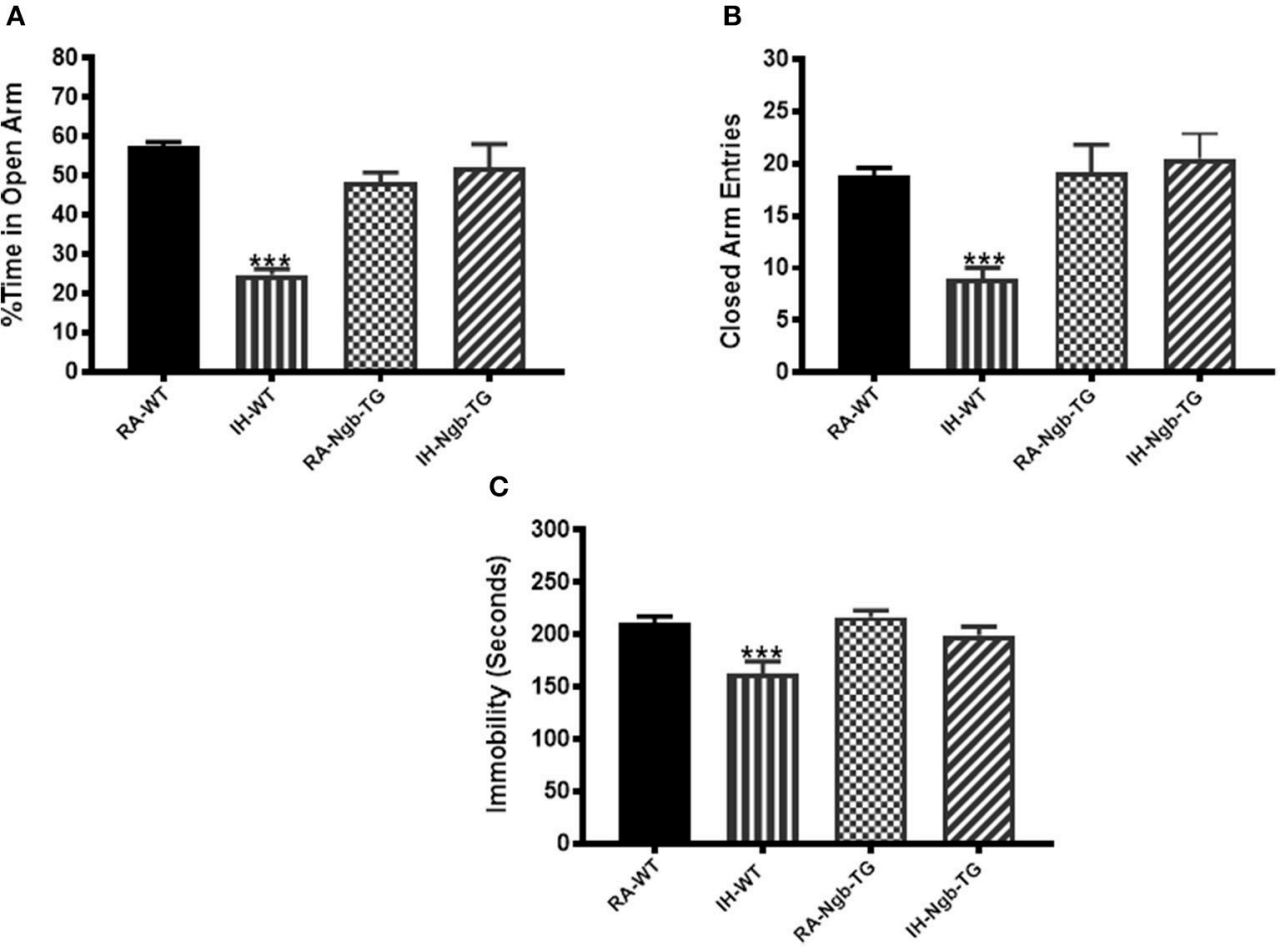
Exposure to IH induces anxiety in mice, which is attenuated in Ngb-TG mice. WT mice exposed to IH spent significantly less time in the open arm of the elevated plus maze compared to RA-WT, or Ngb-TG mice exposed to either RA or IH **(A)**. A reduced number of closed-arm entries emerged in wild type mice exposed to IH **(B)**. (*n* = 14/experimental group; ^***^P < 0.0001 vs. RA-WT) **(C)** Forced-swim test indicates Ngb-TG mice are not depressed following IH exposures. Ngb-TG exposed to IH show less immobility as compared to WT mice exposed to IH. (*n* = 14/experimental group; ^***^P < 0.001 vs. RA-WT).

IH-WT mice displayed significant differences in the percentage of time spent in the open arm (*F* = 17.96; *p* < 0.0001) and in the number of entries into the closed arm (*F* = 8.779; *p* < 0.0001; Figures 5A,B), when compared to all other experimental groups, including IH-Ngb-TG mice.

#### Forced swim test

IH-WT mice exhibited greater immobility periods and cumulative duration during the last 4 min of the FST (*F* = 7.588; *p* < 0.001), when compared to all other treatment groups, including the IH-Ngb-TG mice (Figure 5C).

#### Lipid peroxidation

**Figure 6:**
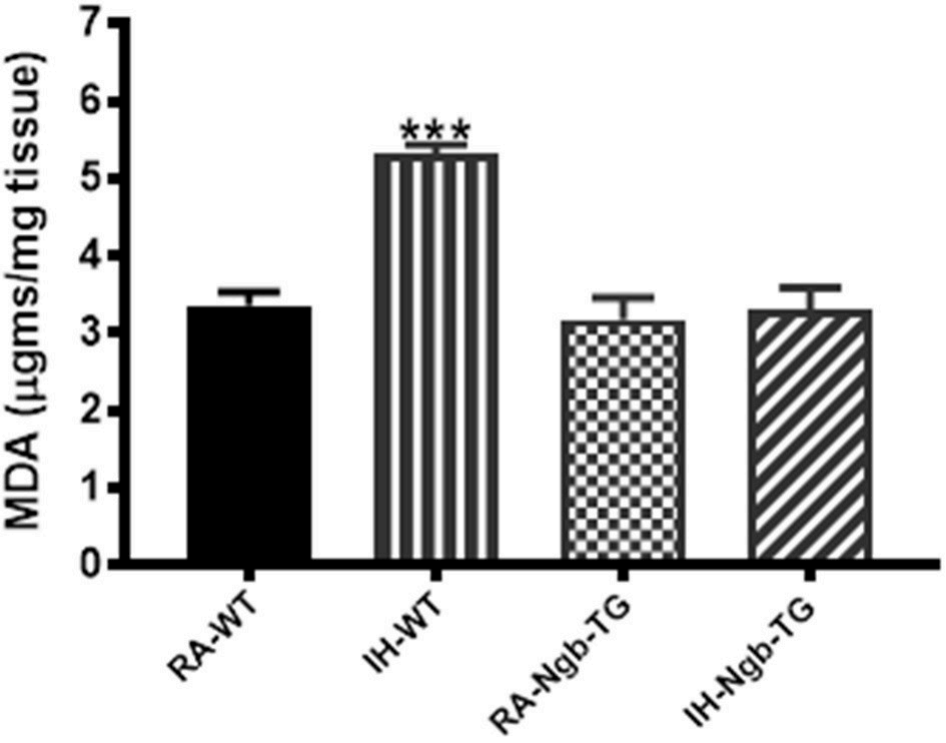
Lipid peroxidation was reduced in the cortex of Ngb-TG mice exposed to IH. MDA tissue levels in cortex of Ngb-TG and WT mice exposed to either room air (RA) or intermittent hypoxia for 14 days (IH). (*n* = 6 per experimental group; ^***^P < 0.0001 vs. RA-WT).

After the behavioral experiments, frontotemporal cortical tissues were harvested and processed for assessment of lipid peroxidation as reported by MDA concentrations. Figure 6 shows MDA levels as measured in homogenates of frontotemporal cerebral cortex from all treatment groups. MDA levels were significantly higher in IH-WT mice (*F* = 20.84; *p* < 0.0001) when compared to all other experimental groups.

## Discussion

This study shows that overexpression of Ngb in mice significantly attenuates cognitive and behavioral deficits induced by long-term exposures to intermittent hypoxia modeling sleep apnea. In addition, Ngb overexpression reduced the degree of brain tissue lipid peroxidation, as reflected by lower MDA levels in the frontotemporal cortex of IH-exposed Ngb mice.

The presence of excessive lipid peroxidation reflects alterations in the brain induced by a variety of disease states including sleep apnea. The CNS is particularly susceptible to lipid peroxidation because it contains an abundance of polyunsaturated fatty acids that serve as the substrate for oxidation reactions. Lipid peroxidation causes structural cell membrane damage and produces diffusible secondary bioactive aldehydes including 4-hydroxy-2-nonenal and acrolein (27), both of which are increased in several brain regions affected by advanced neurodegenerative diseases (28, 29). Keller and colleagues (30) showed that malondialdehyde, thiobarbituric acid–reactive substances, and protein carbonyls were all increased in the superior and middle temporal gyri in patients afflicted with Alzheimer disease or stroke. The systemic oxidative stress response to acute ischemia-reperfusion injury such as in stroke involves increases in 8-OHdG and MDA, which have also been associated with poor functional recovery (31). Studies from our lab in a mouse model of sleep apnea have also reported the presence of increased oxidative stress as induced chronic intermittent hypoxia, and that the excessive lipid peroxidation mediated brain cortical neuronal cell apoptosis (32) and spatial learning deficits (5, 33). Current results further corroborate that IH induces cognitive impairments and concomitant increases in lipid peroxidation in frontotemporal brain tissues in IH-WT mice.

In the nearly 2 decades since its discovery by Burmester and colleagues (34), Ngb has been identified as playing significant functional roles in neuroprotection that clearly transcend its original putative function as an oxygen transporter (35–37). Overexpression of Ngb reduced the extent of brain injury as elicited by experimentally-induced intracerebral hemorrhage (38), while exogenous Ngb protein administration (via treatment with Ngb that was fused to the 11-amino-acid human immunodeficiency virus transactivator of transcription protein transduction domain) not only efficiently transduced into neurons in the mouse brain, but also afforded significant protection to the brain from mild or even moderate ischemic injury (39). However, we are unaware of any studies examining the potential contribution of Ngb in the context of chronic IH exposures such as those occurring in sleep-disordered-breathing. In fact, we showed several years ago that IH differentially promotes the expression of two related oxygen-binding globins, namely Ngb and cytoglobin in brain in a region dependent fashion (7). A study on recombinant cytoglobin gene transfected into SH-SY5Y neuroblastoma cells using lipofectamine exhibited a neuroprotective effect following cobalt chloride-induced hypoxia (40). Furthermore, we showed that compared to prolonged sustained hypoxia (SH), such as encountered during extended sojourns at high altitude, IH of similar duration did not elicit the robust increases in Ngb brain expression that were consistently present following SH (7). The exact mechanisms underlying the failure of IH to induce increases in the expression of Ngb are unclear. However, we have recently shown that hypoxia-inducible factor-1α (HIF-1α) transcriptional activity is markedly reduced, even absent, during chronic IH-exposures compared to SH of similar duration (41). Thus, it is possible that the inability of IH to recruit and induce the expression of neuroprotective elements such as Ngb, while at the same time markedly enhancing oxidative stress (4, 42, 43), may underlie the neuronal susceptibility to IH, ultimately manifesting as cognitive and behavioral deficits. Thus, the current study provides valuable information regarding the protective role of Ngb in the context of IH-induced cognitive deficits. Our current experiments using the Morris Water Maze replicated several of our previous findings, whereby IH-WT mice exhibit substantial reductions in their ability to acquire and retain a spatial task (5, 20–22, 24). In contrast, IH-Ngb-TG mice revealed preservation of spatial learning and memory. The Morris Water Maze is a hippocampal-dependent test of spatial learning and memory, and therefore, the preserved performances of the Ngb-TG mice after IH on the Morris Water Maze likely reflect the attenuation of oxidative stress, the latter being induced by IH (5, 32, 44) and enhanced neuronal survival in the hippocampus. Wakasugi and colleagues (45) proposed a novel role for Ngb as an intrinsic sensor of oxidative stress in brain, based on its structural homology when compared to regulators of G protein signaling (RGS) and RGS domains of G protein-coupled receptor kinases. These investigators found that oxidation of ferrous to ferric iron moieties in Ngb conferred the guanine nucleotide with dissociation inhibitor activity capacity, and as a result, it would activate signaling pathways that promote cell survival (46). Similar such studies have supported a strong correlation between Ngb overexpression and cell survival and protection using Ngb transgenic mice exposed to ischemia-inducing paradigms (16) and to oxidative stress (11) models. More recently, a putative additional role for Ngb in the context of mitochondrial integrity and function has also been advanced (47, 48), and is clearly aligned with the mitochondrial dysfunction in neurons exposed to IH (4, 49, 50).

Clinical studies have shown that patients with OSA also exhibit symptoms of anxiety, depression and mood disturbances (51–54). Evidence for regional brain structural injury, functional alterations, and metabolic deficits occur in limbic regions classically associated with negative emotions among OSA patients. Indeed, brain areas, such as the amygdala, hippocampus, insular, and cingulate cortices are particularly affected (52, 55). These sites underlie components of emotional behaviors such as fear (amygdala, hippocampus) (55, 56) and dyspnea (insula, cingulate cortex) (57, 58). Although we did not specifically and systematically explore all the brain regions underlying the expression of depression or anxiety, we here showed that their manifestation after IH was indeed detectable using standardized and widely accepted behavioral tests, thereby replicating the clinical phenotypes frequently encountered in human patients suffering from OSA. The EPM test is one of the most frequently adopted tests to examine anxiety in rodents (59, 60). The open and closed arms are designed to evoke the innate exploratory drive, and the degree of aversion of mice to explore the open arms of the maze is viewed as reflecting fear of open and elevated spaces, thus as a consequence of the induction of higher levels of anxiety (60, 61). The FST is a depressive-like behavior test using an inescapable stressor paradigm, whereby the adoption by the animal of a passive floating posture in face of the aversive water immersion is viewed as indicative of depressive behaviors, and as such, this test is widely used to evaluate animal models of depression or alternatively the efficacy of antidepressant medications (40, 62). Indeed, FST-associated immobility is considered as a learning process by the exposed animal that the escape from water is impossible, thereby reflecting learned helplessness (63). However, we should also stress that the EPM and the forced swim tests are very different in the functions they portend to examine. Notwithstanding, we also note that aversive stimulation is very important in both tests. Given such stimulus similarity between the tests, the relationship between time in the plus-maze open arms and floating behaviors during the forced swim test could be explained by an overall increased reactivity to aversive stimulation. Reactivity to aversive stimulation is likely involved in anxiety and depression. Stressors (both psychological and physiological) can impact the performance in both EPM and FST tests, and can often do so long after the stressor has ceased. To this effect, we cannot exclude with certainty that the control mice may differ in their EPM and FST responses when exposed to IH because of previous experiences during their initial 5 weeks of life at the vendor facility till their purchase and transfer to our laboratory vivarium. The remarkable similarity between the findings in the WT and Ngb mice exposed to room air conditions somewhat dissuades from such possibility, even if not conclusively. Furthermore, a high rate of co-morbidity exists between patients suffering from anxiety disorders and from depression (64, 65), with the two disorders sharing some overlapping characteristics such as irritability, sleep disturbances, and difficulty concentrating (66). However, in addition to being a central symptom of anxiety disorders, anxiety itself is often a component of the negative affect associated with depression (67, 68). Although anxiety is not explicitly stated in the diagnostic criteria for major depression (66), an exceedingly large proportion of depressed patients report anxiety as a symptom (69). Indeed, anxiety without depression is much more common than depression without anxiety (67, 68). Our findings show that IH exposures modified and enhanced anxiety-like behaviors in WT mice. In contrast, Ngb-TG exposed to IH showed preserved performances in this test, suggesting that regions underlying these behavioral patterns are susceptible to IH, most likely via an oxidative stress pathway. Similar findings during the forced swim test which is believed to reflect a correlate of depression and stress handling in mice (70) among the IH-WT mice may be indicative underlying oxidative stress.

Collectively, these findings provide incremental evidence on a potential role for Ngb-mediated neuroprotection in the context of IH exposures mimicking OSA, and may open opportunities for development of therapeutic targets aimed at preventing end-organ injury in patients with IH insults secondary to severe OSA. Notwithstanding current findings, increased understanding as to the mechanisms that govern Ngb expression in the context of diverse hypoxic stimuli will be critical in such future studies.

## Conclusion

In the present study, our findings clearly corroborate that IH exposures during the sleep period are associated with significant learning and memory impairments, and that the observed deficits are consistent with the possibility that they may involve oxidative stress. Transgenic mice over-expressing neuroglobin exposed to IH exhibited lower levels of lipid peroxidation in frontotemporal cortex along with preserved behavioral performances, thereby providing evidence of a neuroprotective effect.

Future studies aiming extend current findings and identify potential therapeutic targets for the treatment of OSA are warranted.

## Author contributions

DN and VR performed all experiments, analyzed the data and drafted the initial version of the manuscript. DG coordinated the experimental planning, assisted with data analysis and interpretation, and edited and approved the final version of the manuscript.

### Conflict of interest statement

The authors declare that the research was conducted in the absence of any commercial or financial relationships that could be construed as a potential conflict of interest.

## Abbreviations

OSA: obstructive sleep apnea
Ngb: neuroglobin
IH: intermittent hypoxia
HIF1α: hypoxia inducible factor 1α
ROS: reactive oxygen species
RA: room air
EPM: elevated plus maze
FST: forced swimming test
MDA: malondialdehyde
RA-WT: room air wild-type
IH-WT: intermittent hypoxia wild-type
RA-Ngb-TG: room air neuroglobin transgenic
IH-Ngb-TG: intermittent hypoxia neuroglobin transgenic
ANOVA: analysis of variance
SDB: sleep disordered breathing.

## References

[1] Gozal D Daniel JM Dohanich GP. Behavioral and anatomical correlates of chronic episodic hypoxia during sleep in the rat. J Neurosci. (2001) 21:2442–50. 11264318 10.1523/JNEUROSCI.21-07-02442.2001 PMC6762394

[2] Gozal E Row BW Schurr A Gozal D. Developmental differences in cortical and hippocampal vulnerability to intermittent hypoxia in the rat. Neurosci Lett. (2001) 305:197–201. 11403939 10.1016/s0304-3940(01)01853-5

[3] Row BW Kheirandish L Neville JJ Gozal D. Impaired spatial learning and hyperactivity in developing rats exposed to intermittent hypoxia. Pediatr Res. (2002) 52:449–53. 10.1203/00006450-200209000-00024 12193683

[4] Wang Y Zhang SX Gozal D. Reactive oxygen species and the brain in sleep apnea. Respir Physiol Neurobiol. (2010) 174:307–16. 10.1016/j.resp.2010.09.001 20833273 PMC3088760

[5] Nair D Dayyat EA Zhang SX Wang Y Gozal D. Intermittent hypoxia-induced cognitive deficits are mediated by NADPH oxidase activity in a murine model of sleep apnea. PLoS ONE (2011) 6:e19847. 10.1371/journal.pone.0019847 21625437 PMC3100309

[6] Sun Y Jin K Mao XO Zhu Y Greenberg DA. Neuroglobin is up-regulated by and protects neurons from hypoxic-ischemic injury. Proc Natl Acad Sci USA. (2001) 98:15306–11. 10.1073/pnas.251466698 11742077 PMC65025

[7] Li RC Lee SK Pouranfar F Brittian KR Clair HB Row BW. Hypoxia differentially regulates the expression of neuroglobin and cytoglobin in rat brain. Brain Res. (2006) 1096:173–9. 10.1016/j.brainres.2006.04.063 16750520

[8] Hundahl CA Allen GC Hannibal J Kjaer K Rehfeld JF Dewilde S. Anatomical characterization of cytoglobin and neuroglobin mRNA and protein expression in the mouse brain. Brain Res. (2010) 1331:58–73. 10.1016/j.brainres.2010.03.056 20331985

[9] Li RC Guo SZ Lee SK Gozal D. Neuroglobin protects neurons against oxidative stress in global ischemia. J Cereb Blood Flow Metab. (2010) 30:1874–82. 10.1038/jcbfm.2010.90 20571522 PMC3023926

[10] Haines B Demaria M Mao X Xie L Campisi J Jin K. Hypoxia-inducible factor-1 and neuroglobin expression. Neurosci Lett. (2012) 514:137–40. 10.1016/j.neulet.2012.01.080 22342914 PMC3526664

[11] Sun Y Jin K Peel A Mao XO Xie L Greenberg DA. Neuroglobin protects the brain from experimental stroke *in vivo*. Proc Natl Acad Sci USA. (2003) 100:3497–500. 10.1073/pnas.0637726100 12621155 PMC152321

[12] Hundahl CA Fahrenkrug J Hay-Schmidt A Georg B Faltoft B Hannibal J. Circadian behaviour in neuroglobin deficient mice. PLoS ONE (2012) 7:e34462. 10.1371/journal.pone.0034462 22496809 PMC3320642

[13] Chen LM Xiong YS Kong FL Qu M Wang Q Chen XQ. Neuroglobin attenuates Alzheimer-like tau hyperphosphorylation by activating Akt signaling. J Neurochem. (2012) 120:157–64. 10.1111/j.1471-4159.2011.07275.x 21496024

[14] Herold S Fago A Weber RE Dewilde S Moens L. Reactivity studies of the Fe(III) and Fe(II)NO forms of human neuroglobin reveal a potential role against oxidative stress. J Biol Chem. (2004) 279:22841–7. 10.1074/jbc.M313732200 15020597

[15] Khan AA Wang Y Sun Y Mao XO Xie L Miles E. Neuroglobin-overexpressing transgenic mice are resistant to cerebral and myocardial ischemia. Proc Natl Acad Sci USA. (2006) 103:17944–8. 10.1073/pnas.0607497103 17098866 PMC1693852

[16] Wang X Liu J Zhu H Tejima E Tsuji K Murata Y. Effects of neuroglobin overexpression on acute brain injury and long-term outcomes after focal cerebral ischemia. Stroke (2008) 39:1869–74. 10.1161/STROKEAHA.107.506022 18403737 PMC2727360

[17] Sanchez AI Buela-Casal G Bermudez MP Casas-Maldonado F. The effects of continuous positive air pressure treatment on anxiety and depression levels in apnea patients. Psychiatry Clin Neurosci. (2001) 55:641–6. 10.1046/j.1440-1819.2001.00918.x 11737799

[18] Hobzova M Prasko J Vanek J Ociskova M Genzor S Holubova M. Depression and obstructive sleep apnea. Neuro Endocrinol Lett. (2017) 38:343–52. 29106789

[19] Leng Y McEvoy CT Allen IE Yaffe K. Association of sleep-disordered breathing with cognitive function and risk of cognitive impairment: a systematic review and meta-analysis. JAMA Neurol. (2017) 74:1237–45. 10.1001/jamaneurol.2017.2180 28846764 PMC5710301

[20] Li RC Row BW Kheirandish L Brittian KR Gozal E Guo SZ. Nitric oxide synthase and intermittent hypoxia-induced spatial learning deficits in the rat. Neurobiol Dis. (2004) 17:44–53. 10.1016/j.nbd.2004.05.006 15350964

[21] Kheirandish L Row BW Li RC Brittian KR Gozal D. Apolipoprotein E-deficient mice exhibit increased vulnerability to intermittent hypoxia-induced spatial learning deficits. Sleep (2005) 28:1412–7. 10.1093/sleep/28.11.1412 16335482

[22] Kaushal N Ramesh V Gozal D. Human apolipoprotein E4 targeted replacement in mice reveals increased susceptibility to sleep disruption and intermittent hypoxia. Am J Physiol Regul Integr Comp Physiol. (2012) 303:R19–29. 10.1152/ajpregu.00025.2012 22573105 PMC3404642

[23] Gileles-Hillel A Almendros I Khalyfa A Nigdelioglu R Qiao Z Hamanaka RB. Prolonged exposures to intermittent hypoxia promote visceral white adipose tissue inflammation in a murine model of severe sleep apnea: effect of normoxic recovery. Sleep (2017) 40:zsw074. 10.1093/sleep/zsw074 28329220

[24] Gozal D Khalyfa A Qiao Z Almendros I Farre R. Temporal trajectories of novel object recognition performance in mice exposed to intermittent hypoxia. Eur Respir J. (2017) 50:1701456. 10.1183/13993003.01456-2017 29242263

[25] Nair D Zhang SX Ramesh V Hakim F Kaushal N Wang Y. Sleep fragmentation induces cognitive deficits via nicotinamide adenine dinucleotide phosphate oxidase-dependent pathways in mouse. Am J Respir Crit Care Med. (2011) 184:1305–12. 10.1164/rccm.201107-1173OC 21868506 PMC3262045

[26] Gerlai R Clayton NS. Analysing hippocampal function in transgenic mice: an ethological perspective. Trends Neurosci. (1999) 22:47–51. 10.1016/S0166-2236(98)01346-0 10092042

[27] Esterbauer H Schaur RJ Zollner H. Chemistry and biochemistry of 4-hydroxynonenal, malonaldehyde and related aldehydes. Free Radic Biol Med. (1991) 11:81–128. 10.1016/0891-5849(91)90192-6 1937131

[28] Markesbery WR Lovell MA. Four-hydroxynonenal, a product of lipid peroxidation, is increased in the brain in Alzheimer's disease. Neurobiol Aging (1998) 19:33–6. 10.1016/S0197-4580(98)00009-8 9562500

[29] Lovell MA Xie C Markesbery WR. Acrolein is increased in Alzheimer's disease brain and is toxic to primary hippocampal cultures. Neurobiol Aging (2001) 22:187–94. 10.1016/S0197-4580(00)00235-9 11182468

[30] Keller JN Schmitt FA Scheff SW Ding Q Chen Q Butterfield DA. Evidence of increased oxidative damage in subjects with mild cognitive impairment. Neurology (2005) 64:1152–6. 10.1212/01.WNL.0000156156.13641.BA 15824339

[31] Chen YC Chen CM Liu JL Chen ST Cheng ML Chiu DT. Oxidative markers in spontaneous intracerebral hemorrhage: leukocyte 8-hydroxy-2'-deoxyguanosine as an independent predictor of the 30-day outcome. J Neurosurg. (2011) 115:1184–90. 10.3171/2011.7.JNS11718 21962000

[32] Xu W Chi L Row BW Xu R Ke Y Xu B. Increased oxidative stress is associated with chronic intermittent hypoxia-mediated brain cortical neuronal cell apoptosis in a mouse model of sleep apnea. Neuroscience (2004) 126:313–23. 10.1016/j.neuroscience.2004.03.055 15207349

[33] Row BW Liu R Xu W Kheirandish L Gozal D. Intermittent hypoxia is associated with oxidative stress and spatial learning deficits in the rat. Am J Respir Crit Care Med. (2003) 167:1548–53. 10.1164/rccm.200209-1050OC 12615622

[34] Burmester T Weich B Reinhardt S Hankeln T. A vertebrate globin expressed in the brain. Nature (2000) 407:520–23. 10.1038/35035093 11029004

[35] Burmester T Hankeln T. Function and evolution of vertebrate globins. Acta Physiol (2014) 211:501–14. 10.1111/apha.12312 24811692

[36] Guidolin D Tortorella C Marcoli M Maura G Agnati LF. (2016). Neuroglobin, a factor playing for nerve cell survival. Int J Mol Sci. 17:1817. 10.3390/ijms17111817 27809238 PMC5133818

[37] Reeder BJ. Redox and peroxidase activities of the hemoglobin superfamily: relevance to health and disease. Antioxid Redox Signal. (2017) 26:763–76. 10.1089/ars.2016.6803 27637274

[38] Jin K Mao X Xie L Greenberg DA. Neuroglobin expression in human arteriovenous malformation and intracerebral hemorrhage. Acta Neurochir Suppl. (2011) 111:315–9. 10.1007/978-3-7091-0693-8_52 21725774 PMC3234111

[39] Cai B Lin Y Xue XH Fang L Wang N Wu ZY. TAT-mediated delivery of neuroglobin protects against focal cerebral ischemia in mice. Exp Neurol. (2011) 227:224–31. 10.1016/j.expneurol.2010.11.009 21093435

[40] Cryan JF Holmes A. The ascent of mouse: advances in modelling human depression and anxiety. Nat Rev Drug Discov. (2005) 4:775–90. 10.1038/nrd1825 16138108

[41] Gozal D Gileles-Hillel A Cortese R Li Y Almendros I Qiao Z. Visceral white adipose tissue after chronic intermittent and sustained hypoxia in mice. Am J Respir Cell Mol Biol. (2017) 56:477–87. 10.1165/rcmb.2016-0243OC 28107636

[42] Lavie L. Oxidative stress in obstructive sleep apnea and intermittent hypoxia–revisited–the bad ugly and good: implications to the heart and brain. Sleep Med Rev. (2015) 20:27–45. 10.1016/j.smrv.2014.07.003 25155182

[43] Zhou L Chen P Peng Y Ouyang R. Role of oxidative stress in the neurocognitive dysfunction of obstructive sleep apnea syndrome. Oxid Med Cell Longev. (2016) 2016:9626831. 10.1155/2016/9626831 27774119 PMC5059616

[44] Shan X Chi L Ke Y Luo C Qian S Gozal D. Manganese superoxide dismutase protects mouse cortical neurons from chronic intermittent hypoxia-mediated oxidative damage. Neurobiol Dis. (2007) 28:206–15. 10.1016/j.nbd.2007.07.013 17719231 PMC2100412

[45] Wakasugi K Nakano T Morishima I. Oxidized human neuroglobin acts as a heterotrimeric Galpha protein guanine nucleotide dissociation inhibitor. J Biol Chem. (2003) 278:36505–12. 10.1074/jbc.M305519200 12860983

[46] Schwindinger WF Robishaw JD. Heterotrimeric G-protein betagamma-dimers in growth and differentiation. Oncogene (2001) 20:1653–60. 10.1038/sj.onc.1204181 11313913

[47] Yu Z Zhang Y Liu N Yuan J Lin L Zhuge Q. Roles of neuroglobin binding to mitochondrial complex III subunit cytochrome c1 in oxygen-glucose deprivation-induced neurotoxicity in primary neurons. Mol Neurobiol. (2016) 53:3249–57. 10.1007/s12035-015-9273-4 26050086

[48] Chen F Lu J Lin Z Lin Y Yu L Su X. Recombinant neuroglobin ameliorates early brain injury after subarachnoid hemorrhage via inhibiting the activation of mitochondria apoptotic pathway. Neurochem Int. (2018) 112:219–26. 10.1016/j.neuint.2017.07.012 28774717

[49] Douglas RM Ryu J Kanaan A Del Carmen Rivero M Dugan LL Haddad GG. Neuronal death during combined intermittent hypoxia/hypercapnia is due to mitochondrial dysfunction. Am J Physiol Cell Physiol. (2010) 298:C1594–602. 10.1152/ajpcell.00298.2009 20357179 PMC2889641

[50] Wang L Zhang P Wang H Yu J Han X Zhang M. A preliminary study of the effect of mitochondrial autophagy on cognitive function in rats of early intermittent hypoxia. Zhonghua Jie He He Hu Xi Za Zhi (2014) 37:840–44. 25604115

[51] Kumar R Macey PM Cross RL Woo MA Yan-Go FL Harper RM. Neural alterations associated with anxiety symptoms in obstructive sleep apnea syndrome. Depress Anxiety (2009) 26:480–91. 10.1002/da.20531 18828142 PMC4041684

[52] Yaouhi K Bertran F Clochon P Mezenge F Denise P Foret J. A combined neuropsychological and brain imaging study of obstructive sleep apnea. J Sleep Res. (2009) 18:36–48. 10.1111/j.1365-2869.2008.00705.x 19250174

[53] Shapiro AL. Anxiety in middle-aged men with obstructive sleep apnea: state of the science. J Am Assoc Nurse Pract. (2014) 26:689–95. 10.1002/2327-6924.12118 24688014

[54] Kerner NA Roose SP. Obstructive sleep apnea is linked to depression and cognitive impairment: evidence and potential mechanisms. Am J Geriatr Psychiatry (2016) 24:496–508. 10.1016/j.jagp.2016.01.134 27139243 PMC5381386

[55] Hasler G Fromm S Alvarez RP Luckenbaugh DA Drevets WC Grillon C. Cerebral blood flow in immediate and sustained anxiety. J Neurosci. (2007) 27:6313–9. 10.1523/JNEUROSCI.5369-06.2007 17554005 PMC2713601

[56] Williams LM Phillips ML Brammer MJ Skerrett D Lagopoulos J Rennie C. Arousal dissociates amygdala and hippocampal fear responses: evidence from simultaneous fMRI and skin conductance recording. Neuroimage (2001) 14:1070–9. 10.1006/nimg.2001.0904 11697938

[57] Peiffer C Poline JB Thivard L Aubier M Samson Y. Neural substrates for the perception of acutely induced dyspnea. Am J Respir Crit Care Med. (2001) 163:951–7. 10.1164/ajrccm.163.4.2005057 11282772

[58] Evans KC Banzett RB Adams L McKay L Frackowiak RS Corfield DR. BOLD fMRI identifies limbic, paralimbic, and cerebellar activation during air hunger. J Neurophysiol. (2002) 88:1500–11. 10.1152/jn.2002.88.3.1500 12205170

[59] Pellow S Chopin P File SE Briley M. Validation of open:closed arm entries in an elevated plus-maze as a measure of anxiety in the rat. J Neurosci Methods (1985) 14:149–67. 10.1016/0165-0270(85)90031-7 2864480

[60] Rodgers RJ Dalvi A. Anxiety, defence and the elevated plus-maze. Neurosci Biobehav Rev. (1997) 21:801–10. 10.1016/S0149-7634(96)00058-9 9415905

[61] Korte SM De Boer SF. A robust animal model of state anxiety: fear-potentiated behaviour in the elevated plus-maze. Eur J Pharmacol. (2003) 463:163–75. 10.1016/S0014-2999(03)01279-2 12600708

[62] Padilla E Shumake J Barrett DW Sheridan EC Gonzalez-Lima F. Mesolimbic effects of the antidepressant fluoxetine in Holtzman rats, a genetic strain with increased vulnerability to stress. Brain Res. (2011) 1387:71–84. 10.1016/j.brainres.2011.02.080 21376019 PMC3081853

[63] Stedenfeld KA Clinton SM Kerman IA Akil H Watson SJ Sved AF. Novelty-seeking behavior predicts vulnerability in a rodent model of depression. Physiol Behav. (2011) 103:210–16. 10.1016/j.physbeh.2011.02.001 21303678 PMC3925672

[64] Kessler RC Gruber M Hettema JM Hwang I Sampson N Yonkers KA. Co-morbid major depression and generalized anxiety disorders in the National Comorbidity Survey follow-up. Psychol Med. (2008) 38:365–74. 10.1017/S0033291707002012 18047766 PMC2745899

[65] Beesdo K Pine DS Lieb R Wittchen HU. Incidence and risk patterns of anxiety and depressive disorders and categorization of generalized anxiety disorder. Arch Gen Psychiatry (2010) 67:47–57. 10.1001/archgenpsychiatry.2009.177 20048222

[66] American Psychiatric Association (1994). Diagnostic and Statistical Manual of Mental Disorders. 4th ed Washingtion, DC (1994).

[67] Mineka S Watson D Clark LA. Comorbidity of anxiety and unipolar mood disorders. Annu Rev Psychol. (1998) 49:377–412. 10.1146/annurev.psych.49.1.377 9496627

[68] Morilak DA Frazer A. Antidepressants and brain monoaminergic systems: a dimensional approach to understanding their behavioural effects in depression and anxiety disorders. Int J Neuropsychopharmacol. (2004) 7:193–218. 10.1017/S1461145704004080 15003145

[69] Regier DA Rae DS Narrow WE Kaelber CT Schatzberg AF Prevalence of anxiety disorders and their comorbidity with mood and addictive disorders. Br J Psychiatry (1998) 34:24–8. 9829013

[70] Bogdanova OV Kanekar S D'Anci KE Renshaw PF. Factors influencing behavior in the forced swim test. Physiol Behav. (2013) 118:227–39. 10.1016/j.physbeh.2013.05.012 23685235 PMC5609482

